# *Pseudophylloporus* Gen. nov. and *Rubroleccinum* Gen. nov., Two New Genera Revealed by Morphological and Phylogenetic Evidences in the Family Boletaceae from Subtropical China

**DOI:** 10.3390/jof10120817

**Published:** 2024-11-25

**Authors:** Hua-Zhi Qin, Yi Wang, Wen-Fei Lin, Hui Zeng, Li-Gui Hu, Bin-Rong Ke, Zhi-Heng Zeng, Zhi-Qun Liang, Nian-Kai Zeng

**Affiliations:** 1 Ministry of Education Key Laboratory for Ecology of Tropical Islands, Key Laboratory of Tropical Animal and Plant Ecology of Hainan Province, College of Life Sciences, Hainan Normal University, Haikou 571158, China; huazhiqin2022@163.com (H.-Z.Q.); wangy199@foxmail.com (Y.W.); 2 Institute of Edible and Medicinal Fungi, College of Life Sciences, Zhejiang University, Hangzhou 310058, China; 0920158@zju.edu.cn (W.-F.L.); liguihu@163.com (L.-G.H.); 3 Institute of Edible Mushroom, Fujian Academy of Agricultural Sciences, Fuzhou 350011, China; zenghui@faas.cn (H.Z.); kebinrong@163.com (B.-R.K.); zengzhiheng@faas.cn (Z.-H.Z.); 4 School of Pharmacy, Hainan Medical University, Haikou 571199, China; 5 School of Chemistry and Chemical Engineering, Hainan University, Haikou 570228, China

**Keywords:** bolete, molecular phylogeny, morphology, new taxa, taxonomy

## Abstract

Boletaceae, the largest and most diverse family of Boletales (Agaricomycetes and Basidiomycota), is both ecologically and economically important. Although many taxa have been described in China, the diversity of the family still remains incompletely understood. In the present study, *Pseudophylloporus baishanzuensis* gen. nov., sp. nov. and *Rubroleccinum latisporus* gen. nov., sp. nov. are proposed based on morphological and molecular phylogenetic analyses. These findings contribute to a deeper understanding of the diversity within the Boletaceae family.

## 1. Introduction

Boletaceae Chevall., the largest and most diverse family in Boletales (Agaricomycetes and Basidiomycota), has been in the spotlight in mycology [1,2]. With the rapid development of contemporary morphology and molecular phylogenetics, the comprehension of Boletaceae has significantly improved, leading to the discovery of numerous new taxa, especially in Asian and American regions [3,4,5,6,7,8,9,10,11,12,13,14]. Recently, Boletaceae has been divided into eight subfamilies, viz., Austroboletoideae G. Wu & Zhu L. Yang, Boletoideae Singer, Chalciporoideae G. Wu & Zhu L. Yang, Leccinoideae G. Wu & Zhu L. Yang, Phylloboletelloideae Dentinger, Tremble, Halling, T.W. Henkel & Moncalvo, Suillelloideae Dentinger, Tremble, Halling, T.W. Henkel & Moncalvo, Xerocomoideae Singer, and Zangioideae G. Wu, Y. C. Li & Zhu L. Yang [3,15,16,17]. Among these subfamilies, approximately 100 genera and 1200 species have been reported [3,4,14,15,16].

In China, people pay much attention to the family Boletaceae, for a significant number of species possess edibility and medicinal values, leading to considerable economic benefits [3,4,14,15]. Moreover, most species establish ectomycorrhizal symbiotic associations with host plants, including Fagaceae, Pinaceae, Dipterocarpaceae, and Myrtaceae, playing an important role in upholding the diversity and homeostasis of forest ecosystems [1,14,18,19,20,21,22,23]. In the subtropical regions of China, there are considerable forests dominated by Fagaceae trees, which provide good habitats for the growth and reproduction of boletes [3,14,24,25]. Although numerous boletes have been revealed in subtropical China [3,8,15,20], there are still many taxa awaiting to be uncovered. Recently, several collections of Boletaceae were made from the region (Zhejiang and Fujian Provinces); further morphology and molecular phylogenetic analyses confirm that these collections represent two novel genera. They are described in an effort to further demonstrate the diversity of Boletaceae in China.

## 2. Materials and Methods

### 2.1. Morphological Studies

The studied specimens were collected from Zhejiang and Fujian Provinces in China, then dried at 50–60 °C for 12 h and deposited in the Fungal Herbarium of Hainan Medical University (FHMU), Haikou City, Hainan Province of China. Field records and digital photographs of fresh specimens were made. Color documentation of fresh materials followed Kornerup and Wanscher [26]. Micromorphological features were observed and measured by 5% KOH solution or stained with 1% Congo Red. Sections of the pileipellis taken from the pileus between the center and margin, and sections of the stipitipellis were taken from the middle part along the longitudinal axis of the stipe [27,28]. All line drawings of microstructures were drawn by freehand. Basidiospores of dried specimens were examined with a JSM-7100F field emission scanning electron microscope (Tokyo, Japan). The number of measured basidiospores is given as n/m/p, where “n” represents the total number of basidiospores measured from “m” basidiomata of “p” collections. Dimensions of basidiospores were presented in the form (a–) b–e–c (–d), where the range b–c contains at least of 90% of the measured values (5th to 95th percentile), “a” and “d” were the extreme values, and “e” refers to the average length/width of basidiospores. Q refers to the length/width ratio of basidiospores; Qm refers to the average Q of basidiospores and is given with a standard deviation [29,30]. The size of the basidiospore was analyzed using SPSS Statistics Version 17.0 [31]. The terms referring to the size of the basidioma were based on Bas [32].

### 2.2. Molecular Procedures

Total genomic DNA was obtained with the Plant Genomic DNA Kit (KANGWEI Company, Taizhou, China) from materials dried with silica gel according to the manufacturer’s instructions. Fragments of three nuclear loci, including LR0R/LR5 [33,34] for the nuclear ribosomal large subunit RNA (28S), EF1-2F/EF1-2R [20] for the translation elongation factor 1-α gene (*TEF1*), and bRPB2-6F/bRPB2-7.1R [35] for the RNA polymerase II second largest subunit gene (*RPB2*), were used. The polymerase chain reaction (PCR) procedures were executed, referring to Xie et al. [31]. PCR products were checked in 1% (*w*/*v*) agarose gels, and positive reactions with a bright single band were purified and directly sequenced using an ABI 3730xl DNA Analyzer (Guangzhou Branch of BGI, Guangzhou, China) with the same primers used for PCR amplifications. The new generated DNA sequences were compiled using BioEdit v7.0.9 [36] and then uploaded to GenBank.

### 2.3. Dataset Assembly

There were eighteen new generated DNA sequences (six of 28S, six of *TEF1*, and six of *RPB2*) from six collections. For the concatenated dataset, the sequences of 28S, *TEF1*, and *RPB2* from the new specimens were aligned with sequences of taxa from previous studies and GenBank (Table 1). *Phlebopus portentosus* (Berk. & Broome) Boedijn and *Boletinellus merulioides* (Schwein). Murrill were selected as the outgroup. To test for phylogenetic conflict among the different genes in the combined dataset, the phylogenetic trees based on 28S, *TEF1,* and *RPB2* datasets were analyzed and conducted using the ML method to detect the topologies of the genes used. The results of the analyses showed that the different gene fragments were not in conflict. Then, three datasets (28S, *TEF1*, and *RPB2*) were aligned with MUSCLE v3.6 [37] and concatenated using Phyutility v2.2 for further analyses [38].

### 2.4. Phylogenetic Analyses

For the multi-gene (28S + *TEF1* + *RPB2*) phylogenetic analyses, both maximum likelihood (ML) and Bayesian Inference (BI) were conducted. Maximum likelihood tree generation and bootstrap analyses were performed with the program RAxML 7.2.6 [77]. All parameters in the ML analysis were maintained at their default values, except the model set to GTRGAMMA [3]. Nonparametric bootstrapping with 1000 replicates was used to gain statistical support. MrBayes 3.1 was employed to implement the Markov Chain Monte Carlo (MCMC) technique for Bayesian analysis [78]. Two runs were established, each consisting of four chains, with sampling from the posterior distribution occurring every 100 generations. The default values for all other parameters were maintained and complemented in MrModeltest 2.3 [79]. Bayesian analysis of the combined nuclear dataset (28S + *TEF1* + *RPB2*) was run for 40 million generations, and the average deviation of split frequencies was 0.003331. The first 25% generations of trees sampled were discarded as burn-in, and Bayesian posterior probabilities (PP) were then calculated for a majority consensus tree of the retained Bayesian trees. The best fit likelihood model for 28S, *TEF1*, and *RPB2* were GTR + I + G, GTR + I + G, and SYM + I + G, respectively.

## 3. Results

### 3.1. Molecular Data

**Figure 1:**
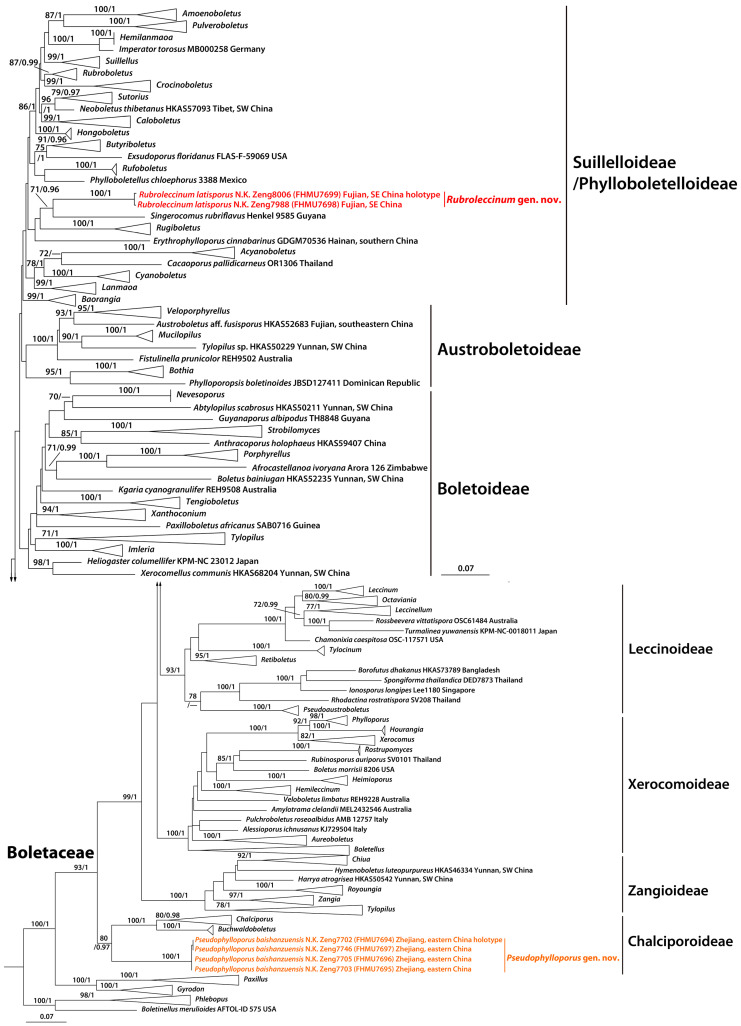
A phylogram of Boletales inferred from a three-locus (28S, *TEF1*, and *RPB2*) dataset using RAxML. BS (≥70%) and PP (≥0.95) are indicated above the branches. Newly generated sequences are in color; SW: southwestern, NE: northeastern, and SE: southeastern.

The combined dataset (28S + *TEF1* + *RPB2*) consisted of 145 sequences with 2329 nucleotide sites, and the alignment was submitted to TreeBASE (S31712). The phylogram with branch lengths generated from RAxML and support values (BS and PP) are shown in Figure 1. The topologies of the phylogenetic trees generated from ML and BI analyses were identical, though statistical support for some branches showed slight differences. The existing molecular data demonstrated that our new collections formed two generic clades within Boletaceae (Figure 1).

### 3.2. Taxonomy

***Pseudophylloporus*** N.K. Zeng, H.Z. Qin, W.F. Lin & L.G. Hu, **gen. nov.**

MycoBank: MB 855763.

**Etymology**—Named because of its phenotypic similarity to the genus *Phylloporus*.

**Diagnosis**—Differs from genera phylogenetically and morphologically close to the new genus by a lamellate hymenophore, lamellae usually forked, a blue-red-black color change of hymenophore and context when injured, smooth basidiospores, and a presence of clamp connections (Table 2).

Basidiomata pileate-stipitate with lamellate hymenophore. Pileus convex to plano-convex; surface dry to slightly viscous, nearly smooth, yellowish-brown to earthy yellow; context white to yellow, turning blue, then changing red, and finally black when injured when injured. Hymenophore lamellate, lamellate usually forked, yellow to yellowish-brown, turning blue, then changing red, and finally black when injured. Stipe central, solid, subcylindrical, base enlarged to subglobose; surface dry, tawny to pale brown, densely covered with pale brown scales; context pale yellow, turning blue, then changing red, and finally black when injured; basal mycelium yellowish. Basidiospores fusoid to elongate, smooth; pleuro- and cheilocystidia present; pileipellis a cutis. Clamp connections present in all tissues.

**Type species**—*Pseudophylloporus baishanzuensis* N.K. Zeng, H.Z. Qin, W.F. Lin & L.G. Hu

*Pseudophylloporus baishanzuensis* N.K. Zeng, H.Z. Qin, W.F. Lin & L.G. Hu, sp. nov.

MycoBank: MB 855764.

**Figure 2:**
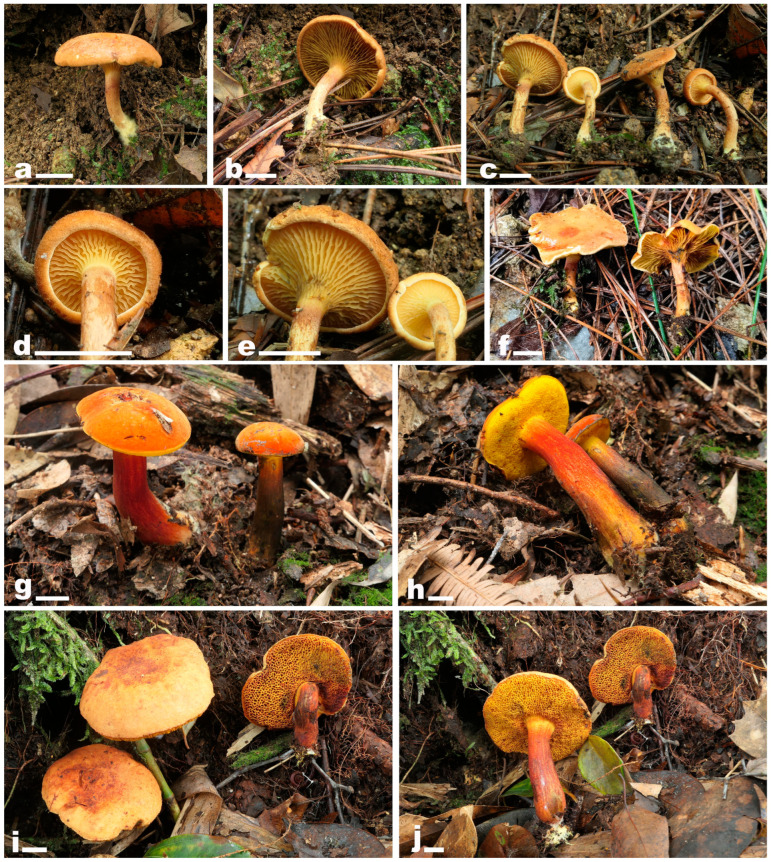
Basidiomata of *Pseudophylloporus* and *Rubroleccinum* species. (**a**–**f**) *Pseudophylloporus baishanzuensis* ((**a**,**b**) from FHMU7694, holotype; (**c**–**e**) from FHMU7695; (**f**) from FHMU7697); (**g**–**j**) *Rubroleccinum latisporus* ((**g**,**h**) from FHMU7698; (**i**,**j**) from FHMU7699, holotype). Scale bars  =  1 cm. Photographs by N.K. Zeng.

**Figure 3:**
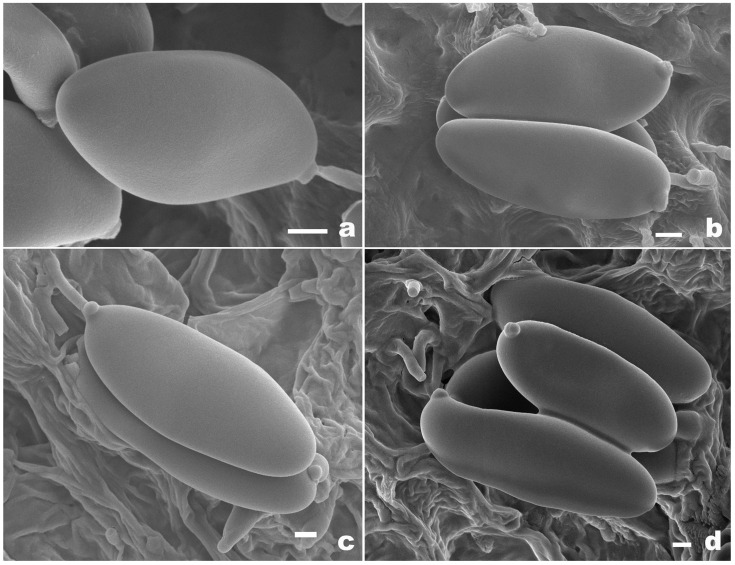
Basidiospores of *Pseudophylloporus* and *Rubroleccinum* species from herbarium materials under SEM. (**a**,**b**) *Pseudophylloporus baishanzuensis* (FHMU7694, holotype); (**c**,**d**) *Rubroleccinum latisporus* (FHMU7699, holotype). Scale bars: 1 μm. Photographs by H.Z. Qin.

**Figure 4:**
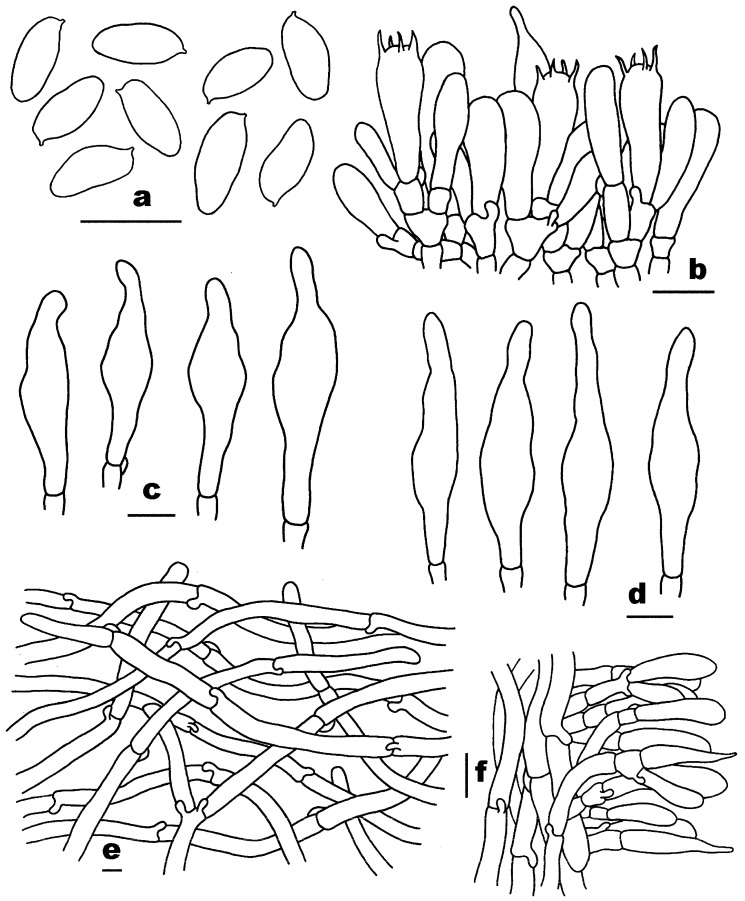
Microscopic features of *Pseudophylloporus baishanzuensis* (FHMU7694, holotype). (**a**) Basidiospores. (**b**) Basidia and pleurocystidium. (**c**) Pleurocystidia. (**d**) Cheilocystidia. (**e**) Pileipellis. (**f**) Stipitipellis. Scale bars = 10 μm. Drawings by H.Z. Qin.

Figure 2a–f, Figure 3a,b and Figure 4.

Etymology—Latin “*baishanzuensis*”, referring to the name of the type locality.

Holotype—CHINA. Zhejiang Province: Lishui City, Qingyuan County, Baishanzu National Forest Park, elev. 1300 m, 12 August 2023, N.K. Zeng7702 (FHMU7694). GenBank accession number: 28S = PQ330210, *TEF1* = PQ330110, *RPB2* = PQ330114.

Diagnosis—The new species is characterized by a blue-red-black color change of hymenophore and context when injured, forked lamellae, yellowish basal mycelia, a cutis pileipellis, and a presence of clamp connections.

Description—Basidiomata is very small to small-sized. Pileus 1.2–3.6 cm diam, convex to plano-convex, becoming applanate with age; surface dry to slightly viscous, smooth, yellowish-brown (5A5), earthy yellow (5A6–5B6) to pale brown (5B5–8); margin incurved, slightly straight when mature; context 0.1–0.6 cm thick in the center of the pileus, white (2A1), pale yellow (2A2) to yellow (2A4), turning blue (24B6), then changing red (10A6), and finally black when injured. Hymenophore lamellate, decurrent; lamellae 0.1–0.3 cm in height, subdistant, usually forked, yellow (1A5–2A5) to yellowish-brown (2B5, 3B5–6), turning blue (24C7) quickly, then changing red (10A6), and finally black (10F7) when injured. Stipe 1.7–3 × 0.3–0.5 cm, central, solid, subcylindrical, base enlarged to subglobose; surface dry, tawny (3A5–3B5) to pale brown (4B4–5), densely covered with pale brown (3B5) sometimes reddish-brown (6B8) scales; context pale yellow (2A2) to yellowish-brown (4A5), turning blue (24B6), then changing red (10A6), and finally black when injured; basal mycelium yellowish (2A4). Odor indistinct.

Basidiospores [160/8/4] 7–9.26–10.5 (–11) × 3–3.99–4 (–5) μm, Q = 2–2.57 (–3), Qm = 2.32 ± 0.16, fusoid to elongate, slightly thick-walled (0.8–1 μm), smooth, pale yellow to yellow in KOH. Basidia 18–33 × 5–9 μm, clavatet o subcylindric, slightly thick-walled (up to 0.8 μm), 4-spored, colorless to yellowish in KOH; sterigmata 2–5 μm in length. Hymenophoral trama composed of slightly thin-walled (up to 0.5 μm) hyphae, 5–15 μm wide, colorless to yellowish in KOH. Pleurocystidia 42–62 × 7–14 μm, subfusiform, slightly thick-walled (up to 1 μm), pale yellow to yellow in KOH, no encrustations. Cheilocystidia 38–74 × 10–15 μm, subfusiform, slightly thick-walled (up to 1 μm), yellow in KOH, no encrustations. Pileipellis a cutis 140–380 μm thick, composed of slightly thick-walled (up to 1 μm) hyphae, subparallel to slightly interwoven, 3–11 μm wide, colorless in KOH; terminal cells 41–119 × 6–14 μm, clavate or subcylindrical. Pileal trama composed of slightly thick-walled (up to 1 μm) hyphae, 8–23 μm wide, colorless in KOH. Stipitipellis a trichoderm-like structure 25–75 μm thick, composed of slightly thick-walled (up to 1 μm) hyphae, 3–9 μm wide, yellowish in KOH; terminal cells 28–39 × 5–7 μm, clavate to subcylindrical. Stipe trama composed of parallel hyphae, slightly thick-walled (up to 1 μm), 5–31 μm wide, subcylindrical, yellowish in KOH. Clamp connections are present in all tissues.

Habitat—Solitary or gregarious on the ground in forests dominated by fagaceous trees.

Known distribution—Eastern China (Zhejiang Province).

Additional specimens examined—CHINA. Zhejiang Province: Lishui City, Qingyuan County, Baishanzu National Forest Park, elev. 1300 m, 12 August 2023, N.K. Zeng7703 (FHMU7695); same location and date, N.K. Zeng7705(FHMU7696); same location and date, N.K. Zeng7746 (FHMU7697).

***Rubroleccinum*** N.K. Zeng, H.Z. Qin & H. Zeng, **gen. nov.**

MycoBank: MB 855749.

**Etymology**—Latin “*Rubro*-” means a stipe punctuated with red scabers, and “-*leccinum*” refers to the morphological similarities of the new genus with leccinoid mushrooms.

**Diagnosis**—Differs from genera phylogenetically and morphologically close to the new genus by a red-tinged basidioma, a stipe punctuated with red to reddish-brown scabers, yellow basal mycelia, a blue-red color change of hymenophore and context when injured, and a trichoderm pileipellis (Table 3).

Basidiomata pileate-stipitate with tubular hymenophore. Pileus convex to plano-convex; surface dry, nearly smooth, reddish-orange to grayish-yellow; context yellow, changing blue, then turning red when injured. Hymenophore brilliant yellow to yellow, changing blue, then turning red when injured. Stipe central, solid, subcylindrical; surface dry, punctuated with red to reddish-brown scabers; context yellow, changing blue, then turning red when injured; basal mycelium yellow. Basidiospores cylindrical to fusoid, smooth; pleuro- and cheilocystidia present; pileipellis is a trichoderm. Clamp connections are absent in all tissues.

**Type species**—*Rubroleccinum latisporus* N.K. Zeng, H.Z. Qin & H. Zeng

***Rubroleccinum latisporus*** N.K. Zeng, H.Z. Qin & H. Zeng, **sp. nov.**

MycoBank: MB 855750.

**Figure 5:**
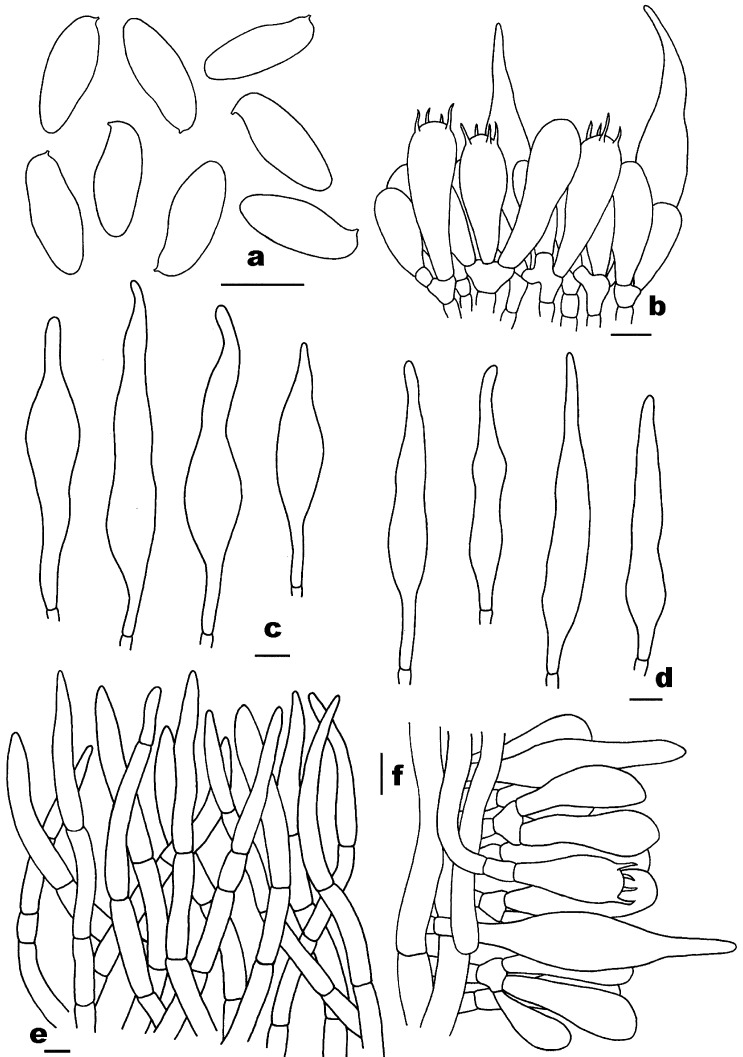
Microscopic features of *Rubroleccinum latisporus* (FHMU7699, holotype). (**a**) Basidiospores. (**b**) Basidia and pleurocystidia. (**c**) Pleurocystidia. (**d**) Cheilocystidia. (**e**) Pileipellis. (**f**) Stipitipellis. Scale bars = 10 μm. Drawings by H.Z. Qin.

Figure 2g–j, Figure 3c,d and Figure 5.

**Etymology**—Latin “*latisporus*” refers to the wide basidiospores.

**Holotype**—CHINA. Fujian Province: Wuyishan City, Wuyi Mountain National Forest Park, elev. 1100 m, 17 August 2023, N.K. Zeng8006 (FHMU7699). GenBank accession number: 28S = PQ325254, *TEF1* = PQ330107, *RPB2* = PQ330109.

**Diagnosis**—The new species is characterized by a red-tinged basidioma, a stipe punctuated with red to reddish-brown scabers, yellow basal mycelia, a blue-red color change of hymenophore and context when injured, wide basidiospores, and a trichodermal pileipellis with cuspidal apex of terminal cells.

**Description**—Basidiomata very small to medium-sized. Pileus 2–5.5 cm diam, subhemispherical when young, then convex to plano-convex; surface dry, nearly smooth, orange (5A7–8) to reddish-orange (6A8) when young, then grayish-yellow (4A3–4) to reddish-brown (6B8–7C7); margin incurved; context 0.5–1.25 cm thick in the center of the pileus, yellow (3A5–7), changing blue (24D7), then turning red when injured. Hymenophore poroid, depressed around apex of stipe, slightly decurrent; pores angular to subround, brilliant yellow (2A6–7) to yellow (4A6), changing blue (24D7), then turning red when injured; tubes 0.4–3 cm in length, yellow (4A6), changing blue (24D7), then turning red when injured. Stipe 2.9–5.1 × 0.5–1.5 cm, central, solid, subcylindrical; surface dry, yellow (4A6), punctuated with red (9A7–8) to reddish-brown (8C7–8) scabers; context yellow (3A6), changing blue (24D7), then turning red when injured; basal mycelium yellow (2A4). Odor indistinct.

Basidiospores [80/4/2] 12.5–14.44–16 (–17.5) × 5–5.67–6 μm, Q = (2.17–) 2.27–2.83 (–3.1), Qm = 2.56 ± 0.18, cylindrical to fusoid, slightly thick-walled (up to 1 μm), smooth, pale yellow to yellowish brown in KOH. Basidia 28–43 × 10–14 μm, subclavate or subcylindric, thin- to slightly thick-walled (0.5–0.8 μm), 4-spored, yellowish in KOH; sterigmata 2–7 μm in length. Hymenophoral trama composed of slightly thick-walled (up to 1 μm) hyphae, 4–13 μm wide, colorless to yellowish in KOH. Pleurocystidia 58–106 × 11–17 μm, abundant, subfusiform, thin- to slightly thick-walled (0.5–0.8 μm), pale yellow to yellow in KOH, no encrustations. Cheilocystidia 39–100 × 7–14 μm, abundant, subfusiform, thin- to slightly thick-walled (0.5–1 μm), yellowish to yellow, or colorless in KOH, no encrustations. Pileipellis a trichoderm 100–200 μm thick, composed of thin- to slightly thick-walled (0.5–1 μm) hyphae, 4–10 μm wide, pale yellow to yellow in KOH, usually with granular contents; terminal cells 35–77 × 5–11 μm, clavate to subcylindrical, with cuspidal apex. Pileal trama composed of slightly thick-walled (up to 1 μm) hyphae, 4–12 μm wide, yellowish in KOH. Stipitipellis a trichoderm-like structure 170–400 μm thick, composed of slightly thick-walled (up to 1 μm) hyphae, 3–9 μm wide, yellowish in KOH; terminal cells 40–102 × 4–8 μm, subcylindrical to cylindrical. Stipe trama composed of parallel hyphae, slightly thick-walled (0.8–1 μm), 4–13 μm wide, subcylindrical, yellowish in KOH. Clamp connections absent in all tissues.

**Habitat**—Solitary or gregarious on the ground in forests dominated by fagaceous trees.

**Known distribution**—Southeastern China (Fujian Province).

**Additional specimen examined**—CHINA. Fujian Province: Wuyishan City, Wuyi Mountain National Forest Park, elev. 1100 m, 17 August 2023, N.K. Zeng7988 (FHMU7698).

## 4. Discussion

The phylogenetic analyses showed that the new genus *Pseudophylloporus* is a member of subfamily Chalciporoideae within Boletaceae (Figure 1). The lamellate hymenophore of *Pseudophylloporus* is reminiscent of several other genera, viz., *Phylloporus* Quél., *Phyllobolites* Singer, *Phylloboletellus* Singer, *Phylloporopsis* Angelini, A. Farid, Gelardi, M.E. Smith, Costanzo, & Vizzini, *Erythrophylloporus* Ming Zhang & T.H. Li, and *Paxilloboletus* Furneaux, De Kesel & F.K. Khan. *Phylloporus,* a genus of subfamily Xerocomoideae, morphologically differs from *Pseudophylloporus* by usually not forked lamellae, basidiospores with bacillate ornamentation, and clamp connections usually absent [4,5,20]. *Phyllobolites,* a genus with undefined subfamily ranking, is different by a membranous ring deriving from a partial veil, a pileal context unchanging or changing blue when injured, a hymenophore turning sienna or rust-color to chestnut when injured, verrucose basidiospores with slight rugulose, and an absence of clamp connections [12,80,81]. *Phylloboletellus*, a member of subfamily Phylloboletelloideae, can be distinguished from *Pseudophylloporus* by a blue (without red) color change of hymenophore and context when injured; basidiospores with longitudinal, continuous, or bifurcate ribs; and sometimes scarce clamp connections [12,81,82]. *Phylloporopsis*, a genus of subfamily Austroboletoideae, is characterized by a hymenophore sometimes sub-boletinoid, lamellae usually not forked, a blue (without red) color change of the hymenophore and context when injured, and an absence of clamp connections [12]. *Erythrophylloporus*, a member of subfamily Suillelloideae, differs from *Pseudophylloporus* by an orange, reddish-orange to yellowish-red basidioma, a red hymenophore, lamellae usually not forked, a vivid yellow to orange yellow context changing dark violet to blackish blue when injured, and an absence of clamp connections [51]. *Paxilloboletus*, a member of subfamily Boletoideae, is distinguished from *Pseudophylloporus* by all tissues unchanging in color when injured and an absence of clamp connections [13].

Phylogenetically, *Pseudophylloporus* is closely related to *Buchwaldoboletus* Pilát and *Chalciporus* Bataille (Figure 1). However, *Buchwaldoboletus* has a poroid hymenophore, a blue (without red) color change of hymenophore and context when injured, an absence of clamp connections, and usually a saprophytic habit [39]. *Chalciporus* differs from *Pseudophylloporus* by a poroid hymenophore, hymenophore and context unchanging or turning bluish when injured, and an absence of clamp connections [39]. The molecular data indicated that the new genus *Rubroleccinum* is assigned to the subfamily Suillelloideae, which has been recognized as an independent subfamily based on the whole genome sequences [16]. Despite the inability to differentiate the subfamilies Suillelloideae and Phylloboletelloideae based on our multi-locus (28S + *TEF1* + *RPB2*) phylogenetic analysis, it is sufficient to demonstrate that *Rubroleccinum* exhibits distinct phylogenetic variations (Figure 1).

The obvious scabers on stipe of *Rubroleccinum* are reminiscent of several other genera, viz., *Hemileccinum* Šutara, *Leccinellum* Bresinsky & Manfr. Binder, *Leccinum* Gray, and *Sutorius* Halling, Nuhn & N.A. Fechner. *Hemileccinum*, a member of subfamily Xerocomoideae, can be distinguished from *Rubroleccinum* by a tissue unchanging in color when injured, irregularly warty basidiospores, and a hyphoepithelium pileipellis [4,68,83,84]. *Leccinellum*, a genus of subfamily Leccinoideae, is characterized by a whitish or yellow hymenophore unchanging or staining brownish to ferruginous, or at first reddish then blackish when injured, scabrous squamules over the surface of stipe brown to blackish, and an epithelium pileipellis [85,86]. *Leccinum*, also a member of subfamily Leccinoideae, has a whitish or yellow hymenophore, a white to cream context unchanging or staining blue or red when injured, and scabrous to dotted squamules on the stipe brown to blackish [15,87,88,89,90]. *Sutorius*, also a genus of subfamily Suillelloideae, differs from *Rubroleccinum* by a hymenophore dark purple, purplish red or purplish brown, and a context without color change or staining blue to dark blue when injured [39,44,91,92].

Phylogenetically, *Rubroleccinum* is closely related to *Singerocomus* T.W. Henkel & M.E. Sm. (Figure 1). However, *Singerocomus* can be distinguished from *Rubroleccinum* by all tissues unchanging in color when injured [7].

In the Boletaceae, abundant taxa exhibit poroid hymenophore [1,2,3,4,7,8,9]. However, more and more boletes with lamellate hymenophore have been discovered recently, and these genera are distributed across different subfamilies within Boletaceae (Figure 1), which indicated that the trait of lamellate hymenophore is a multiple occurrence event evolutionarily [4,5,12,13,20,51,80,81,82].

In the subtropical regions of China, there is a rich diversity of Boletaceae species [1,2,3,4]. Among them, many boletes such as *Butyriboletus* spp. and *Neoboletus* spp. are commercially traded for edibility, which are contributing to economic benefits [6,8,15,39,93,94]. It is noteworthy that the consumption records for the newly identified genera *Rubroleccinum* and *Pseudophylloporus* have not been documented at a collected location. Further studies including toxicity assessments of the two genera should be conducted. Although the edibility of *Rubroleccinum* and *Pseudophylloporus* remains unclear, they are symbiotic with trees of Fagaceae, influencing the growth and nutrient uptake of trees and other ecological processes [1,95,96,97]. Revealing the ecological roles of *Rubroleccinum* and *Pseudophylloporus* is also an interesting study, which enhances our understanding of the complexity and stability of ecological networks, facilitating more effective conservation efforts for subtropical forests of China.

## 5. Conclusions

Although abundant taxa of Boletaceae have been revealed, the diversity of this family has not been completely resolved. In this work, *Pseudophylloporus baishanzuensis* gen. nov., sp. nov. and *Rubroleccinum latisporus* gen. nov., sp. nov. are described based on morphological and molecular phylogenetic analyses. These findings contribute to a deeper understanding of the diversity within the Boletaceae family.

## Figures and Tables

**Table 1:** Taxa, vouchers, locations, and GenBank accession numbers of DNA sequences used in this study.

Taxon	Voucher	Locality	GenBank Accession Nos.			Reference
			28S	*TEF1*	*RPB2*	
*Abtylopilus scabrosus*	HKAS50211	Yunnan, SW China	KT990552	KT990752	KT990389	[39]
*Acyanoboletus controversus*	HKAS126560	Yunnan, SW China	OQ888714	OQ873451	OQ873490	[3]
*Acyanoboletus dissimilis*	ZT14030	Malaysia	OQ888716	OQ873453	OQ873492	[3]
*Afrocastellanoa ivoryana*	Arora 126	Zimbabwe	KX685721	KX685715	—	[40]
*Alessioporus ichnusanus*	KJ729504	Italy	KJ729504	KJ729513	—	[41]
*Amoenoboletus miraculosus*	ZT14046	Malaysia	MW520188	MW566745	—	[18]
*Amoenoboletus* *granulopunctatus*	HKAS56280	Yunnan, SW China	KF112418	KF112265	KF112708	[15]
*Amylotrama clelandii*	MEL2432546	Australia	MT459235	MN413630	—	[42]
*Anthracoporus holophaeus*	HKAS59407	China	KT990708	KT990888	KT990506	[39]
*Aureoboletus catenarius*	HKAS54467	Yunnan, SW China	KT990510	KT990711	KT990349	[39]
*Aureoboletus duplicatoporus*	HKAS83115	Yunnan, SW China	KT990512	KT990713	KT990351	[39]
*Austroboletus* aff. *fusisporus*	HKAS52683	Fujian, SE China	KF112484	KF112213	KF112766	[15]
*Baorangia rufomaculata*	4414	USA	KF030248	KF030406	—	[43]
*Baorangia pseudocalopus*	HKAS75739	Yunnan, SW China	KJ184558	KJ184570	KM605179	[44]
*Boletellus* aff. *emodensis*	HKAS 52678	Fujian, SE China	KF112426	KF112305	KF112757	[15]
*Boletellus indistinctus*	HKAS77623	Guangdong, southern China	KT990531	KT990733	KT990371	[39]
*Boletinellus merulioides*	AFTOL-ID 575	USA	AY684153	DQ056287	—	[45]
*Boletus bainiugan*	HKAS52235	Yunnan, SW China	KF112457	KF112203	KF112705	[15]
*Boletus morrisii*	8206	USA	KF030326	KF030433	—	[43]
*Borofutus dhakanus*	HKAS73789	Bangladesh	JQ928616	JQ928576	JQ928597	[46]
*Bothia castanella*	MB03_053	USA	DQ867117	KF030421	—	[43,47]
*Bothia fujianensis*	HKAS82694	Fujian, SE China	KM269193	KM272860	—	[48]
*Buchwaldoboletus lignicola*	HKAS76674	Heilongjiang, NE China	KF112350	KF112277	KF112819	[15]
*Buchwaldoboletus lignicola*	HKAS84904	Germany	KT990538	KT990740	KT990377	[39]
*Butyriboletus pseudospeciosus*	HKAS63513	Yunnan, SW China	KT990541	KT990743	KT990380	[39]
*Butyriboletus regius*	HKAS84878	Germany	MT264910	MT269659	MT269661	Unpublished
*Butyriboletus subsplendidus*	HKAS50444	Yunnan, SW China	KT990540	KT990742	KT990379	[39]
*Cacaoporus pallidicarneus*	OR1306	Thailand	—	MK372272	MK372285	[10]
*Caloboletus calopus*	HKAS74739	Yunnan, SW China	KF112335	KF112166	KF112667	[15]
*Caloboletus yunnanensis*	HKAS63040	Yunnan, SW China	KJ605676	KJ619471	KT990395	[39,49]
*Chalciporus piperatus*	HKAS84882	Germany	KT990562	KT990758	KT990397	[39]
*Chalciporus rubinelloides*	HKAS74952	Yunnan, SW China	KT990565	KT990761	KT990400	[39]
*Chamonixia caespitosa*	OSC-117571	USA	MK601731	MK721085	MK766293	[50]
*Chiua olivaceoreticulata*	HKAS59706	Yunnan, SW China	KT990593	KT990787	KT990428	[39]
*Chiua virens*	HKAS76678	Sichuan, SW China	KF112438	KF112272	KF112793	[15]
*Crocinoboletus laetissimus*	HKAS50232	China	KT990567	KT990762	—	[39]
*Crocinoboletus rufoaureus*	HKAS59820	Hainan, southern China	KF112434	—	KF112709	[15]
*Cyanoboletus brunneoruber*	HKAS80579-1	Yunnan, SW China	KT990568	KT990763	KT990401	[39]
*Cyanoboletus sinopulverulentus*	HKAS59609	Yunnan, SW China	KF112366	KF112193	KF112700	[15]
*Erythrophylloporus cinnabarinus*	GDGM70536	Hainan, southern China	MH374045	MH378802	MH374035	[51]
*Exsudoporus floridanus*	FLAS-F-59069	USA	OL960488	OL960496	OL960503	[52]
*Fistulinella prunicolor*	REH9502	Australia	JX889648	JX889690	MG212630	[53,54]
*Guyanaporus albipodus*	TH8848	Guyana	HQ161868	—	LC043083	[7,55]
*Gyrodon lividus*	REG Gl1	Germany	—	GU187701	GU187786	[56]
*Gyrodon* sp.	HKAS59448	Yunnan, SW China	KF112349	KF112276	KF112818	[15]
*Harrya atrogrisea*	HKAS50542	Yunnan, SW China	KT990694	KT990880	KT990499	[39]
*Heimioporus japonicus*	HKAS52237	Yunnan, SW China	KF112347	KF112228	KF112806	[15]
*Heimioporus subretisporus*	HKAS80582	Yunnan, SW China	KT990574	KT990770	KT990409	[39]
*Heliogaster columellifer*	KPM-NC 23012	Japan	KX685724	KX685718	—	[40]
*Hemilanmaoa retistipitatus*	HMJAU 60052 (H3624)	Guizhou, SW China	OP380695	OP495816	OP495814	[1]
*Hemilanmaoa retistipitatus*	HMJAU 60053 (H3633)	Guizhou, SW China	OP380696	OP495817	OP495815	[1]
*Hemileccinum impolitum*	HKAS84869	Germany	KT990575	KT990771	KT990410	[39]
*Hemileccinum rugosum*	HKAS84355	Yunnan, SW China	KT990578	KT990774	KT990413	[39]
*Hongoboletus ventricosus*	HKAS59660	Yunnan, SW China	KF112358	KF112153	KF112664	[15]
*Hongoboletus ventricosus*	HKAS63598	Yunnan, SW China	OQ888735	KF112152	KF112663	[3,15]
*Hourangia cheoi*	HKAS52269	Yunnan, SW China	KF112385	KF112286	KF112773	[15]
*Hourangia cheoi*	HKAS74774	Yunnan, SW China	KF112384	KF112285	KF112772	[15]
*Hymenoboletus luteopurpureus*	HKAS46334	Yunnan, SW China	KF112471	KF112271	KF112795	[15]
*Imleria badia*	HKAS74714	Germany	KC215212	KC215242	—	[57]
*Imleria obscurebrunnea*	HKAS52557	Yunnan, SW China	KC215220	KC215243	KC215234	[57]
*Imperator torosus*	MB000258	Germany	—	MW566748	MW560082	[18]
*Ionosporus longipes*	Lee1180	Singapore	—	MT085471	MH712031	[58,59]
*Kgaria cyanogranulifer*	REH9508	Australia	JX889646	JX889688	OR263680	[53,60]
*Lanmaoa angustispora*	HKAS74752	Yunnan, SW China	KM605139	KM605154	KM605177	[44]
*Lanmaoa asiatica*	HKAS54094	Yunnan, SW China	KF112353	KF112161	KF112682	[15]
*Leccinellum corsicum*	Buf4507	USA	KF030347	KF030435	—	[43]
*Leccinellum* sp.	HKAS53427	Hunan, central China	KF112488	KF112253	—	[15]
*Leccinum quercinum*	HKAS63502	Yunnan, SW China	KF112444	KF112250	KF112724	[15]
*Leccinum scabrum*	HKAS56371	Yunnan, SW China	KT990587	KT990782	KT990423	[39]
*Leccinum variicolor*	HKAS57758	Yunnan, SW China	KF112445	KF112251	KF112725	[15]
*Mucilopilus castaneiceps*	HKAS71039	Japan	KT990547	KT990748	KT990385	[39]
*Mucilopilus castaneiceps*	HKAS50338	Yunnan, SW China	KT990555	KT990755	KT990391	[39]
*Neoboletus thibetanus*	HKAS57093	Tibet, SW China	KF112326	—	KF112655	[15]
*Nevesoporus nigrostipitatus*	VIES 9901383	Brazil	OM068918	OM160562	—	[11]
*Nevesoporus nigrostipitatus*	VIES 9901384	Brazil	OM068919	—	—	[11]
*Octaviania japonimontana*	KPM-NC-0017812	Japan	JN378486	JN378428	–	[61]
*Octaviania tasmanica*	OSC132097	Australia	JN378494	JN378435	–	[61]
*Paxilloboletus africanus*	SAB0716	Guinea	MZ702479	MZ707865	MZ707869	[13]
*Paxillus rubicundulus*	Ve08.2h10	France	—	KF261553	—	[62]
*Paxillus ammoniavirescens*	Pou09.1	France	—	KF261533	—	[62]
*Paxillus involutus*	SCV11.1	France	—	KF261544	—	[62]
*Phlebopus portentosus*	Php1	Africa	AF336260	GU187735	GU187801	[56,63]
*Phlebopus portentosus*	FHMU5935	Yunnan, SW China	MW783432	MW897345	—	[64]
*Phylloboletellus chloephorus*	3388	Mexico	DQ534658	—	—	[65]
*Phylloporopsis boletinoides*	JBSD127411	Dominican Republic	MH571711	MH588312	—	[12]
*Phylloporus maculatus*	HKAS56683	Yunnan, SW China	JQ967210	JQ967167	—	[20]
*Phylloporus rubrosquamosus*	HKAS 54559	Yunnan, SW China	JQ967219	JQ967175	—	[20]
*Porphyrellus castaneus*	HKAS52554	Yunnan, SW China	KT990697	KT990883	KT990502	[39]
*Porphyrellus porphyrosporus*	HKAS76671	Jilin, NE China	KF112482	KF112243	KF112718	[15]
*Pseudoaustroboletus valens*	HKAS52603	Yunnan, SW China	KM274869	KM274877	—	[66]
*Pseudoaustroboletus valens*	HKAS82644	China	—	MT110359	MT110431	[67]
***Pseudophylloporus*** ***baishanzuensis***	**N.K. Zeng7702 (FHMU7694)**	**Zhejiang, eastern China**	**PQ330210**	**PQ330110**	**PQ330114**	**This study**
***Pseudophylloporus*** ***baishanzuensis***	**N.K. Zeng7703 (FHMU7695)**	**Zhejiang, eastern China**	**PQ330211**	**PQ330111**	**PQ330115**	**This study**
***Pseudophylloporus*** ***baishanzuensis***	**N.K. Zeng7705 (FHMU7696)**	**Zhejiang, eastern China**	**PQ330212**	**PQ330112**	**PQ330116**	**This study**
***Pseudophylloporus*** ***baishanzuensis***	**N.K. Zeng7746 (FHMU7697)**	**Zhejiang, eastern China**	**PQ330213**	**PQ330113**	**PQ330117**	**This study**
*Pulchroboletus roseoalbidus*	AMB 12757	Italy	KJ729499	KJ729512	—	[41]
*Pulveroboletus brunneopunctatus*	HKAS55369	Yunnan, SW China	KT990620	KT990814	KT990455	[39]
*Pulveroboletus mirus*	HKAS57628	Yunnan, SW China	KT990618	KT990812	KT990453	[39]
*Retiboletus brunneolus*	HKAS52680	Fujian, SE China	KF112424	KF112179	KF112690	[15]
*Retiboletus fuscus*	HKAS63590	Yunnan, SW China	KF112417	KF112178	KF112691	[15]
*Rhodactina rostratispora*	SV208	Thailand	—	MG212606	MG212646	[54]
*Rossbeevera vittatispora*	OSC61484	Australia	JN378506	JN378446	—	[61]
*Rostrupomyces sisongkhramensis*	SV0155	Thailand	—	OP358324	OP358316	[68]
*Rostrupomyces sisongkhramensis*	SV0219	Thailand	—	OP358325	OP358317	[68]
*Royoungia boletoides*	Trappe 27456	Australia	JX889655	JX889696	—	[53]
*Royoungia palumanus*	REH9421	Australia	JX889675	JX889685	—	[53]
*Rubinoboletus rubinus*	AF2835	Belgium	—	KT824028	KT823995	[69]
*Rubinosporus auriporus*	SV0101	Thailand	—	MZ355902	MZ355904	[70]
*Rubroboletus latisporus*	HKAS80358	Chongqing, SW China	KP055023	KP055020	KP055029	[71]
*Rubroboletus sinicus*	HKAS68620	Yunnan, SW China	KF112319	KF112146	KF112661	[15]
***Rubroleccinum latisporus***	**N.K. Zeng7988 (FHMU7698)**	**Fujian, SE China**	**PQ325253**	**PQ330106**	**PQ330108**	**This study**
***Rubroleccinum latisporus***	**N.K. Zeng8006 (FHMU7699)**	**Fujian, SE China**	**PQ325254**	**PQ330107**	**PQ330109**	**This study**
*Rufoboletus hainanensis*	KUN-HKAS 59814	Hainan, southern China	KF112336	KF112199	KF112699	[15]
*Rufoboletus hainanensis*	N.K. Zeng2418 (FHMU2437)	Hainan, southern China	KU961652	KU961656	KX453856	[72]
*Rugiboletus brunneiporus*	HKAS83209	Tibet, SW China	KM605134	KM605144	KM605168	[44]
*Rugiboletus extremiorientalis*	HKAS74754	China	KT990639	KT990832	KT990469	[39]
*Singerocomus rubriflavus*	Henkel 9585	Guyana	LC043093	MH645597	—	[7,10]
*Spongiforma thailandica*	DED7873	Thailand	EU685108	KF030436	—	[43,73]
*Strobilomyces atrosquamosus*	HKAS55368	Yunnan, SW China	KT990648	KT990839	KT990476	[39]
*Strobilomyces seminudus*	HKAS59461	Yunnan, SW China	KF112479	KF112260	KF112815	[15]
*Suillellus amygdalinus*	NY00035656	USA	KT990650	KT990840	KT990477	[39]
*Suillellus subamygdalinus*	HKAS57953	Tibet, SW China	KT990652	KT990842	—	[39]
*Sutorius brunneissimus*	HKAS57451	Yunnan, SW China	KM605137	KM605149	KM605172	[44]
*Sutorius brunneissimus*	HKAS50538	Yunnan, SW China	KM605138	KM605150	KM605173	[44]
*Sutorius hainanensis*	HKAS59469	Yunnan, SW China	KF112359	KF112175	KF112669	[15]
*Tengioboletus glutinosus*	HKAS53425	Hunan, central China	KF112341	KF112204	KF112800	[15]
*Tengioboletus reticulatus*	HKAS53426	Hunan, central China	KF112491	KF112313	KF112828	[15]
*Turmalinea yuwanensis*	KPM-NC-0018011	Japan	KC552046	KC552089	—	[74]
*Tylocinum griseolum*	HKAS52612	Yunnan, SW China	KT990631	KT990825	—	[39]
*Tylocinum griseolum*	HKAS50281	Yunnan, SW China	KF112451	KF112284	KF112730	[15]
*Tylopilus* aff. *chromapes*	01-513	Zambia	JX889672	JX889682	—	[53]
*Tylopilus* aff. *virens*	01-541	Zambia	JX889677	JX889687	—	[53]
*Tylopilus otsuensis*	HKAS50240	Yunnan, SW China	KT990553	KT990753	MT110417	[39,67]
*Tylopilus* sp.	HKAS50229	Yunnan, SW China	KF112423	KF112216	KF112769	[15]
*Tylopilus violaceobrunneus*	HKAS89443	Shandong, eastern China	KT990702	KT990886	KT990504	[39]
*Veloboletus limbatus*	REH9228	Australia	—	MN413636	MT747397	Unpublished
*Veloporphyrellus pseudovelatus*	HKAS52258	Yunnan, SW China	JX984540	JX984551	MT110439	[67,75]
*Veloporphyrellus velatus*	HKAS63668	Hainan, southern China	JX984546	JX984554	—	[75]
*Xanthoconium affine*	NY00815399	USA	KT990661	KT990850	KT990486	[39]
*Xanthoconium porophyllum*	HKAS90217	Guangdong, southern China	KT990662	KT990851	KT990487	[39]
*Xerocomellus communis*	HKAS68204	Yunnan, SW China	KT990671	KT990859	KT990495	[39]
*Xerocomus subparvus*	HKAS53387	Fujian, SE China	KF112397	KF112297	KF112788	[15]
*Xerocomus yunnanensis*	HKAS68420	Yunnan, SW China	KT990690	KT990877	—	[39]
*Zangia olivaceobrunnea*	HKAS52275	Yunnan, SW China	HQ326947	HQ326875	—	[76]
*Zangia roseola*	HKAS75046	Yunnan, SW China	KF112414	KF112269	KF112791	[15]

New sequences are shown in bold; SW: Southwestern, NE: Northeastern, SE: Southeastern.

**Table 2:** Comparison of the genera morphologically and phylogenetically related to *Pseudophylloporus*.

Genus	Pileal Surface	Context	Hymenophore	Stipe Surface	Basidiospores	Clamp Connections
*Buchwaldoboletus*	Tomentose or pulverulent, yellow to brownish.	Changing blue when injured.	Poroid, light yellow to ochraceous yellow, changing bluish to dark blue when injured.	Yellow to brown tones, without squamules.	Subfusiform, smooth.	Absence.
*Chalciporus*	Glabrous to obscurely, subtomentose, pinkish-red to reddish-brown.	Unchanging or turning bluish when injured.	Poroid, pinkish red to reddish brown, unchanging or staining bluish to dull blue slowly when injured.	Yellow to brown tones, without squamules.	Subfusiform, smooth.	Absence.
*Erythrophylloporus*	Pruinose or velutinous, subtomentose to faintly squamulose, orange, reddish-orange to yellowish-red.	Changing dark violet to blackish blue when injured.	Lamellate, red, usually not forked, changing grayish blue when injured.	Reddish-orange to yellowish-red, covered with reddish-orange to orange red pruinose scales.	Broadly ellipsoid, ellipsoid to nearly ovoid, smooth.	Absence.
*Paxilloboletus*	Tomentose, white, cream to yellowish.	Changing blue when injured.	Lamellate, yellow to yellowish-brown, regularly bifurcating and anastomosing, unchanging in color when injured.	White to yellowish-white tomentose, with or without ridges or reticulation on its uppermost part.	Ellipsoid to fusiform, smooth.	Absence.
*Phylloboletellus*	Glabrous, yellow-orange to orange-reddish or brown.	Changing blue when injured.	Lamellate, yellowish-green to olive-brown, usually forked, changing blue when injured.	Yellow to mustard yellow, covered with brownish clumps or fibrils.	Broadly ellipsoid, with longitudinal, continuous or bifurcate ribs.	Sometimes scarce.
*Phyllobolites*	Glabrous to tomentose, olivaceous brown or reddish-brown.	Unchanging or changing blue when injured.	Lamellate, sometimes forked, yellowish-cream, yellow to luteous, turning sienna or rust-color to chestnut when injured.	Yellow tone, pulverulent to tomentose.	Fusiform, verrucose.	Absence.
*Phylloporopsis*	Velvety-tomentose to fibrillose, carmin red, dull red to reddish-brown.	Changing blue when injured.	Lamellate, sometimes sub-boletinoid, usually not forked, beige to olive cream, changing blue when injured.	Yellowish-brown to reddish-brown, pruinose to longitudinally fibrillose.	Ellipsoid to fusiform, smooth.	Absence.
*Phylloporus*	Tomentose, yellowish-brown to reddish-brown.	Unchanging in color when injured.	Lamellate, usually not forked, yellow to golden yellow, unchanging or changing blue to greenish-blue when injured.	Yellowish-brown to reddish-brown, tomentose.	Fusoid to fusiform, with bacillate ornamentation.	Usually absent.
*Pseudophylloporus*	Nearly smooth, yellowish-brown to earthy yellow.	Turning blue, then changing red, and finally black when injured.	Lamellate, yellow to yellowish-brown, usually forked, turning blue, then changing red, and finally black when injured.	Tawny to pale brown, densely covered with pale brown scales.	Fusoid to elongate, smooth.	Present.

**Table 3:** Comparison of the genera morphologically and phylogenetically related to *Rubroleccinum*.

Genus	Pileal Surface	Context	Hymenophore	Stipe Surface	Basidiospores	Pileipellis
*Hemileccinum*	Smooth to subtomentose, or rugose, yellowish-brown to reddish-brown.	Unchanging in color when injured.	Poroid, light yellow to olive yellow, unchanging in color when injured.	Whitish, to cream, pale yellow-brown to dark brown, ornamented with white to yellow, or brown scales.	Subfusiform, irregularly warty.	Hyphoepithelioid type.
*Leccinellum*	Glabrous to subtomentose, rugulose or pitted brown, reddish-brown, brown to dark brown.	Changing red when injured.	Poroid, whitish or yellow, unchanging or staining brownish to ferruginous, or at first reddish then blackish when injured.	White, grayish-brown to yellowish-brown, covered with scabrous, brown to blackish squamules.	Subfusiform to ellipsoid, smooth.	Epithelioid type.
*Leccinum*	Glabrous to subtomentose, grayish-white, yellowish-brown to dark brown.	Unchanging or staining blue or red when injured.	Poroid, whitish or yellow, unchanging or staining blue or red when injured.	White, grayish to blackish-brown covered with scabrous to dotted and brown to blackish squamules.	Subfusiform, smooth.	Trichodermal type.
*Rubroleccinum*	Nearly smooth, orange to reddish-orange when young, then grayish-yellow to reddish-brown.	Changing blue, then turning red when injured.	Poroid, brilliant yellow to yellow, changing blue, then turning red when injured.	Yellow to brown, punctuated with red to reddish-brown scabers.	Cylindrical to fusoid, smooth.	Trichodermal type.
*Singerocomus*	Tomentose, pinkish red to red.	Unchanging in color when injured.	Poroid, yellow, unchanging in color when injured.	Yellow to brown, glabrous or with dull yellow squamules.	Ellipsoidal, smooth.	Trichodermal type.
*Sutorius*	Glabrous to subtomentose, chocolate brown to reddish brown or purplish brown.	Unchanging or staining blue to dark blue when injured.	Poroid, dark purple, purplish red or purplish brown, unchanging or turning red when injured.	Pinkish, yellow to reddish-brown, covered with pale brown to brown, or pale light purple squamules.	Subcylindrical to subfusiform, smooth.	Trichodermal type.

## Data Availability

The datasets presented in this study have been deposited in NCBI GenBank (https://www.ncbi.nlm.nih.gov/genbank/) and Mycobank (https://www.mycobank.org/page/Home/MycoBank).
